# Nonprotein Structures from Mycobacteria: Emerging Actors for Tuberculosis Control

**DOI:** 10.1155/2012/917860

**Published:** 2012-05-07

**Authors:** Luz M. Lopez-Marin

**Affiliations:** Centro de Fisica Aplicada y Tecnologia Avanzada, Universidad Nacional Autonoma de Mexico, Campus Juriquilla, Carretera Queretaro-San Luis Potosi km 15.5, 76230 Juriquilla, QRO, Mexico

## Abstract

Immune response to *Mycobacterium tuberculosis*, the causal agent of tuberculosis, is critical for protection. For many decades, consistent to classical biochemistry, most studies regarding immunity to the tubercle bacilli focused mainly on protein structures. But the atypical, highly impermeable and waxy coat of mycobacteria captured the interest of structural biologists very early, allowing the description of amazing molecules, such as previously unknown carbohydrates or fatty acids of astonishing lengths. From their discovery, cell wall components were identified as important structural pillars, but also as molecular motifs able to alter the human immune response. Recently, as new developments have emerged, classical conceptions of mycobacterial immune modulators have been giving place to unexpected discoveries that, at the turn of the last century, completely changed our perception of immunity *vis-à-vis* fat compounds. In this paper, current knowledge about chemical and ultrastructural features of mycobacterial cell-wall is overviewed, with an emphasis on the relationships between cell-wall nonpeptide molecules and immune response. Remarks regarding the potential of these molecules for the development of new tools against tuberculosis are finally discussed.

## 1. Introduction

In 1882, Robert Koch described *Mycobacterium tuberculosis*, the causal agent of tuberculosis to the monthly meeting of the Berlin Physiological Society. The bacilli were extremely difficult to stain for viewing at the microscope, and Koch had to use an ammonia solution for damaging the cell wall and enabling the colorant to stain the cell [1]. Some 50 years after that, before the discovery of streptomycin by Selman Waksman and Albert Schatz, a whole set of antibiotics had been shown to fail for entering the waxy capsule of the tubercle bacillus. In fact, the extremely impermeable coat of *M. tuberculosis* constituted a subject of pessimism within most microbiologists in regards to the search for a drug against tuberculosis. The particular nature of *M. tuberculosis *cell wall was therefore quickly recognized and took the attention from structural biochemists. As the central role of immune response for tuberculosis control was realized, studies were focused on the relationship between host immunity and cell wall compounds. A variety of biochemical and molecular approaches were used to test the involvement of specific carbohydrates and lipids from mycobacterial cell wall in immunogenicity, virulence, and persistence [2–4]. Mycobacteria are known for both activating and downmodulating the immune response, and cell-wall compounds have proved to induce disparate activities towards immunity as well. Today, the *M. tuberculosis* envelope has been definitively recognized as a key factor of pathogenicity, but also as a powerful source of compounds against the disease [4–6]. This paper presents an overview of the structural features of mycobacterial cell wall, and its activities towards the host immune response. In accordance with the evolutionary knowledge about nonprotein compounds of the tubercle bacilli, the importance of these previously neglected molecules for the development of tools against tuberculosis will be highlighted.

## 2. *Mycobacterium tuberculosis* Cell Wall: The Extraordinary Permeability Barrier as Described by Structural Biologists

### 2.1. The Covalently Linked Components: A Cell Wall with Uncommon Constituents

Some 80 years ago, Rudolph J. Anderson initiated at Yale University the study of mycobacterial cell wall. With the search for bacterial sterols in mind, Anderson used a set of chemical reactions and solvent equilibria, but unexpected, interesting facts were evidenced instead [7, 8]. During the second moiety of the last century, various tools for biochemistry research were developed, for instance, the use of enzymes, spectroscopies, mass spectrometry, and chromatographies. Then, the analysis of mycobacterial cell wall became a fascinating field which captured the attention of various structural biologists [7, 9]. Analyses of saccharide cell-wall polymers showed that, in addition to peptidoglycan, a second polysaccharide, the arabinogalactan, featured all mycobacterial species [10]. Arabinogalactan is characterized by atypical cyclic configuration for their monosaccharide units (in a 5-members ring) [11] and is acylated to extremely heavy fatty chains. Only after prolonged saponification, mycobacterial cell wall could liberate fatty acids with skeletons composed of up to 88 carbons, that is, the heaviest fatty compounds that have ever been known in nature, the so-called mycolic acids [12]. Mycolic acids constitute an essential part of mycobacterial cell wall and are species-specific. This specificity has been exploited for the use of mycolic acids as biomarkers, for instance, for the authentication of *M. tuberculosis* in ancient human remains [13]. The extreme chain length of mycolic acids determines the waxy nature of mycobacterial cell wall and has been found to be critical for mycobacterial virulence. For example, genetic manipulation to avoid full elongation of mycolic acids in *M. smegmatis* has resulted in the dramatic loss of drug and temperature resistance in mutant strains [14].

**Figure 1:**
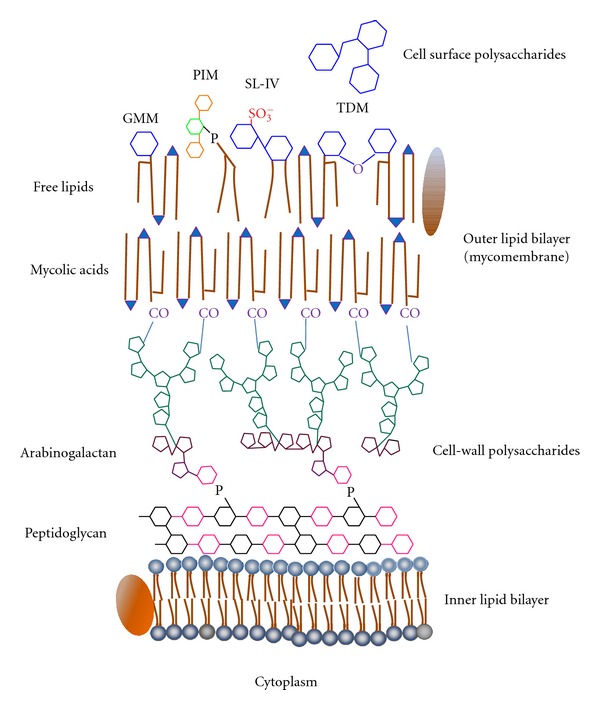
Proposed ultrastructural array of *M. tuberculosis* cell wall. Cell wall includes a great variety of lipids (depicted in brown, with polar functional groups in blue) and sugars (schematized by hexagons and pentagons in different colors). Surrounding the plasma membrane, cell-wall peptidoglycan is attached to another polysaccharide, the arabinogalactan. Long-chain fatty acids, the mycolic acids, are attached to arabinogalactan through ester linkages. An outer lipid bilayer called mycomembrane is formed by mycolic acids and free amphipathic lipids. Finally, some strains display free saccharides, such as glucan, at the outermost layer. GMM, glucose monomycolate; PIM, phosphatidylinositol mannoside; SL, sulfatide; TDM, trehalose dimycolate or cord factor. Cell-wall proteins are depicted in arbitrary positions. Figure is out of scale.

Ultrastructure of mycobacterial cell wall is not affordable by conventional methods; therefore, the spatial location of many lipid components in mycobacterial envelope is still mere speculation. The 3D proposed models for *M. tuberculosis* cell wall are controversial [15, 16]. However, recent data support a folded configuration of mycolic chains, which are thought to form an outer membrane-like structure, reminiscent of Gram-negative bacterial cell walls and called mycomembrane [16–18]. A model showing the essential constituents of *M. tuberculosis* cell wall is depicted in Figure 1.

### 2.2. The Releasable, Free Cell-Wall Compounds

**Figure 2:**
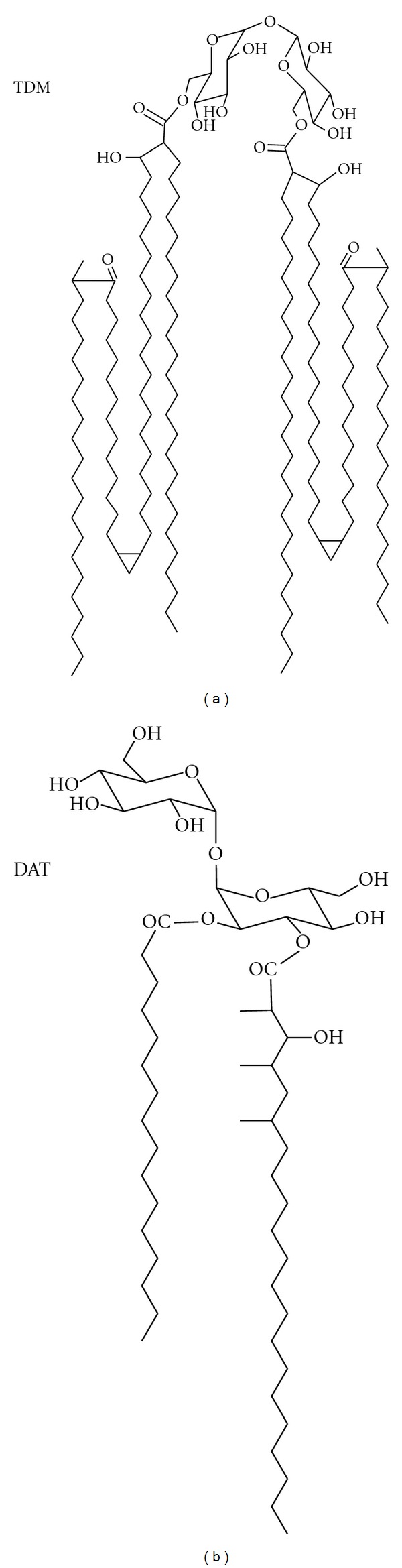
Acylated trehaloses of *M. tuberculosis* cell walls. Acylated variants of alpha,alpha′-(1→1′)-glucosyl glucose (trehalose) are abundant in mycobacterial envelopes. These are free amphipathic lipids known to display paradoxical immune modulatory activities. For instance, trehalose 6,6′-dimycolate (TDM) presents potent proinflammatory and granulomatogenic properties [24, 25], whereas 2,3-di-*O*-acyl trehalose (DAT) exhibits down-modulatory effects on immune cells [26, 27].

The typical motifs in mycobacterial cell walls are not limited to covalently-linked structures. A large proportion of mycobacterial lipid motifs is attached via a noncovalent linkage to the mycolyl-arabinogalactan-peptidoglycan (mAGP) core. These “free” lipids are mostly amphipathic and probably arrange into an outer leaflet in the outermost bilayer of the cell wall (Figure 1). Free mycobacterial lipids were firstly described by Anderson, Lederer, and the Asselineaus [7]. Many free lipids are conjugated to sugar moieties, therefore constituting amphiphilic glycolipids or hydrophilic lipopolysaccharides. For instance, phospholipids linked to inositol and mannoses are typical of all mycobacterial species. These compounds, called phosphatidylinositol mannosides (PIMs), may also contain successive additions of mannose or arabinose residues, forming lipopolysaccharide molecules. Lipomannan (LM) and lipoarabinomannan (LAM), two of the hallmark structures in mycobacteria, belong to this molecular class [9]. Curiously, the precise location of LM and LAM on cell-wall ultrastructure is still to be defined. Acylated glucose or trehalose also typify mycobacterial lipids. Trehalose, a disaccharide composed of two units of D-Glucose coupled through an alpha,alpha′-(1→1′)-linkage, may be esterified by mycolates or other species-specific fatty acids (Figure 2) [12]. Other acyl trehaloses display additional sugars or a sulphate substituent. The major glucose-containing lipids in *M. tuberculosis* are trehalose-6,6′-dimycolate (TDM or cord factor), glucose monomycolate (GMM), 2,3-di-*O*-acyl trehalose (DAT), 2′-sulphate,2,3′-di-*O*-acyl trehalose or diacylated sulphatide (SL) and lipooligosaccharides (LOS) [12, 19].

### 2.3. The Whole Envelope Components in Mycobacteria Form a Unique Cell Wall

Combined, the unusual sugars of the cell wall and the free lipid components (linked through non-covalent interactions) form a unique structure within the microbial world.

Mycobacterial free lipids represent a large and complex set of molecules. Some of them are widespread within the genus, whereas others are composed by a common core motif and differ from one another by fine structural variations that typify species or genotypes [12, 19–21]. Due to this diversity, free glycolipids form distinct arrays in different *M. tuberculosis* strains, giving rise to specific interactions between host cells and bacteria. For instance, some strains display cell-surface polysaccharides hindering free lipids of the mycomembrane. One of these saccharides is glucan, a glucose-containing polymer known to limit opsonic phagocytosis [3] (see Figure 1).

After mycobacterial internalization, noncovalently linked lipids are released from phagocytic vesicles and exported in exosomes to bystander noninfected cells [22, 23]. Implications of this lipid release on mycobacterial virulence and host response will be discussed below. 

## 3. Antigens and Immune Modulators from the Cell Wall: A Biophysicist Point of View

The relationship between mycobacteria and its host is strongly dependant on the establishment of immune mechanisms. Activation of immune effectors, namely, T-cells, is a key step for tuberculosis control. *Mycobacterium tuberculosis* is a powerful pathogen, able to evade the immune machinery and survive inside the macrophage and other phagocytic and nonphagocytic cells [4]. A balanced activation of immune cells allows a protective response to be attained in 90–95% of infected people. In these individuals, *M. tuberculosis* is contained inside a granuloma, whose maintenance depends on the correct function of immune system. Failure to establish an immune response against the pathogen results in the development of active tuberculosis, characterized by an increase in bacterial load. To face the pathogen, which is commonly established in lungs, the immune system guides a complex set of pro-inflammatory mechanisms; however, these may turn to exacerbated responses and lead to pathology and necrosis [4]. *M. tuberculosis* is therefore able to both activate and suppress immunity, a dualistic scheme that determines the fate of tuberculosis infection.

When isolated, cell-wall compounds have been shown to trigger a set of biological effects including adjuvanticity [4, 7], toxicity [24, 25], immune downmodulation [2, 26–29], and arrest of phagosome maturation [30]. Cell-wall moieties, including carbohydrates and lipids, are able to elicit the production of antibodies as well. Many carbohydrate- and lipid-containing molecules are located at the outermost layer of mycobacterial cell envelopes [19], that is, at the site of early events in host-pathogen interactions.

Lipid moieties in the tubercle bacillus are extremely abundant, with some 30% of *M. tuberculosis* genes devoted to lipid synthesis or metabolism [31]. Lipid compounds have been shown to induce many biological activities. For example, the glycolipid dimycoloyl trehalose (DMT), or cord factor, induces tissue damage and necrosis when injected in a murine model [24, 25]; using *in vitro* assays, phenolic glycolipids, typical of some *M. tuberculosis* genotypes, have been found to suppress T-cell proliferation, independently of their fine structural and antigenic features [32]; another cell envelope glycolipid, the phosphatidyl-inositol-mannoside (PIM), has been found to arrest phagosome maturation *in vitro* [30].

In contrast to protein molecules, the effects caused by lipids are not always associated to specific mechanisms of action. Thus, different, unspecific alterations have been proposed to explain the various biological activities on immune cells. Amphiphilic lipids from mycobacterial envelopes were proposed to be released from the infecting bacilli, followed by their integration into host cell membranes, with subsequent alterations on membrane topography and function [33, 34]. In support to this hypothesis, Beatty et al. showed that, following a short incubation period, free lipids are readily transferred from *M. tuberculosis* infected to bystander noninfected cells [22]. Moreover, studies addressing the effects of mycobacterial lipids on liposomes or mammalian cells revealed that lipids induce a set of biological effects, including inhibition of membrane fusion by dimycoloyl trehalose (DMT), increase of membrane permeability, and uncoupling of membrane mitochondrial potential [33].

At present, biological effects caused by various lipids can be explained, at least in part, by newly discovered mechanisms, such as signaling through the binding to innate immune receptors (see below). Notwithstanding, it is to note that free lipids have the potential to be integrated into host cell membranes, so the possibility of unspecific alterations by mycobacterial lipids leading to immune modulation is still an open question. In accordance to this idea, supramolecular architecture in immune cells, (namely, raft domains) induced during cell activation processes, is known to be dependent on membrane lipid composition [35]. Moreover, raft organization in macrophages has been known to be altered after incubation with mycobacterial extractable lipids [36]. 

## 4. Mycobacterial Molecular Patterns: Microbial Sensing through Innate Immune Mechanisms

*M. tuberculosis* infections are mostly contained by the immune system, leading to latent tuberculosis. Notwithstanding, a restricted set of individuals are believed to efficiently eliminate live *M. tuberculosis*, avoiding the establishment of the pathogen either in the latent or in the active form of the infection [4]. In agreement with this hypothesis, some healthy individuals in close contact with *M. tuberculosis* contagious patients fail to show anti-*M. tuberculosis* reactive cells, as indicated by *in vitro* and *in vivo* tests. For this to occur, potent innate immune mechanisms must take place after the ingestion of *M. tuberculosis*, and mycobacterial cell-surface glycoconjugates are probably at the origin of those interactions [4]. Most times, however, *M. tuberculosis* cell entry is followed by a long-term host-pathogen relationship, where various cell types may act as favorable niches.

### 4.1. Phagocytosis through Sugar Receptors

A critical step for mycobacterial fate is the route of cell entry into phagocytic cells. Myeloid-derived cells are able to recognize the pathogen through phagocytic receptors. Complement receptors (CRs) and Fc receptors (FcRs) account within the most important phagocytic receptors in myeloid cells, promoting phagocytosis of complement or immunoglobulin opsonized bacteria. However, primary interactions between mycobacteria and phagocytes may bypass common opsonizing phagocytosis [3]. *M. tuberculosis* strains may expose glucan, a cell-surface polymer structurally similar to human glycogen [3]. According to binding experiments using CR3 expressed in transfected Chinese hamster ovary cells, D-glucan interacts through the lectin site of CR3, thus promoting a particular entry pathway [21]. Other virulent *M. tuberculosis* strains expose mannose-rich glycoconjugates, including lipoarabinomannan (LAM), phosphomannoinositides (PIM), and lipomannan (LM). Recent works by Schlesinger group have demonstrated that ManR, in macrophages, and DC-SIGN, in dendritic cells, importantly regulate the host response against *M. tuberculosis. *Furthermore, sugar recognition through these C-type lectins has been found to be altered according to the degree of saccharide acylation, thus influencing the whole fate of the bacilli [37–39]. Altogether, these data suggest that fine structural variations in mycobacterial molecular patterns are very important to determine the phagocytic pathways.

In addition to sensing by phagocytic receptors, mycobacterial sugars have also been known to bind collectins, a family of proteins phagocytosed through the macrophage CR3 lectin domain. To this family belong the mannose binding lectin (MBL) and the pulmonary surfactant proteins SP-A and SP-B, all of them involved in the recognition of mannose-containing molecules [40].

### 4.2. Host-Pathogen Communication through Nonphagocytic Recognizing Receptors

Some fifteen years ago, studies about *Toll*, a gene involved in the development of *Drosophila melanogaster* embryos, unexpectedly revealed the association of *Toll* to innate immunity in adult flies [41, 42]. The Toll protein homolog in humans, now called as Toll-like receptor 1 (TLR-1), was recognized as an important element of innate immunity, and 10 more TLRs have been described to date [43]. TLRs are transmembrane receptors which sense pathogens at the cellular surface. Evolutionarily conserved for hundreds of millions years, TLRs recognize pathogen-associated molecular patterns, including heat-shock proteins, bacterial CpG motifs, and molecules characterizing bacterial cell envelopes, namely, amphipathic lipids [43]. In particular, TLR-2 has been found to sense mycobacterial lipids, such as LAM, PIMs, and the 19 kDa lipid-containing protein LpqH, whose fatty motif has been shown to be essential for TLR recognition [43].

To accomplish their protective role, TLR members are activated by specific microbial patterns, and trigger signal transduction pathways in cooperation with various signaling adaptors. Some signals are only activated by a cooperative interaction between two TLR molecules or TLRs and another lipid receptor, such as CD14. TLR signaling pathways may involve different mediators, leading to distinct immune responses depending on the molecular signal. Frequently, TLR signaling involves the activation of MAPK pathways, which culminate in the translocation of transcription factors, including NF-*κ*B and activator protein-1 (AP-1), and the expression of mediators for antimycobacterial activities, such as phagolysosome maturation, autophagy, apoptosis, and antigen processing and presentation [43, 44]. However, TLR-2 activation has also been shown to promote opposite, anti-inflammatory functions. In cooperation with *M. tuberculosis* secreted protein ESAT-6, TLR-2 downmodulates the myeloid-differentiation-factor-88 (MyD88-) dependent proinflammatory signaling and may also prevent, in macrophages, upregulation of antigen presenting molecules induced by cytokines [4]. Some studies have suggested that after a prolonged contact with mycobacterial ligands, TLR-2-dependent immune downmodulation occurs, which might reflect a mechanism of negative-feedback regulation in order to prevent excessive inflammation [28, 29].

More recently, additional actors of the innate immune response have been found to detect *M. tuberculosis* cell-wall products. Intracellular proteins which highly resemble plant *R* (resistance) proteins, the nucleotide binding and oligomerization domain (NOD)-like receptors (NLRs), have been shown to sense a variety of products that have been released inside the cell, thereby promoting inflammatory processes against foreign bodies in the cytoplasm, including bacterial molecules. In particular, NOD2 receptor, has been known to sense a peptidoglycan moiety from the cell wall structure typifying mycobacteria [45].

Noteworthy, the description of TLRs and NLRs brought to light how a set of inflammatory processes take place in response to microbes, including mycobacteria, and to various inorganic signals. For instance, the study of NLRs enlightened how mycobacterial cell-wall components, including Freund adjuvants, may induce pro-inflammatory activity. In this regard, deeper studies of inflammatory processes induced by mycobacterial TLRs and NLRs ligands would represent valuable clues for the development of new vaccines against tuberculosis.

Innate immune response to mycobacterial nonprotein molecules probably involves still unknown receptors and modes of action. Two years ago, for instance, Ishikawa and coworkers described Mincle, a lectin type receptor expressed in stressed macrophages, which senses cord factor through an unorthodox way. Mincle has been shown to activate immunity through recognition of 6,6′-di-*O*-acyl trehalose structures, although trehalose alone does not compete for binding [46]. Given the strong relationship now recognized between innate and adaptive immunity, it is probable that more powerful protective vaccines, that include lipid subunits, can be developed during the following years. 

## 5. T-Cell Recognition of Nonprotein Antigens

Adaptive immunity is crucial for protection against *M. tuberculosis*. The effectors of adaptive immunity are T lymphocytes, which are able to recognize bacterial antigens presented by antigen-presenting molecules. For many decades, presentation to T-cells was thought to be limited to peptide antigens. In fact, the expression of peptide presenting molecules, namely, Major Histocompatibility Complex class I and class II molecules (MHCI and MHCII), has represented the hallmark of myeloid-derived mononuclear cells following activation.

For almost a century, live attenuated mycobacteria have been used as vaccines in order to induce adaptive immune responses. A large variety of protein antigens from the tubercle bacilli have been shown to stimulate the immune system, and some of them have been used for the development of acellular vaccine subunits [47]. At the end of the last century, however, the dogma postulating proteins as the unique antigenic molecules driving clonal selection and proliferation of T-cells were reversed by the recognition of both novel antigen-presenting molecules and nonprotein lipid antigens. In fact, the study of the cytotoxic mechanisms involved in a particular autoimmune disease allowed the description of the cluster of differentiation 1 (CD1) molecule as an antigen-presenting molecule [48]. CD1 molecules fundamentally differ from their MHC homologues in the ability to present nonprotein, lipid molecules [49, 50]. Hydrophobic and deep grooves characterize CD1 molecules, which have been shown to bind amphipathic lipids and present them to specific T-cells. Humans express five CD1 isoforms (CD1a, b, c, d, and e), which are distributed at the plasma membrane as well as at the endocytic pathway. Both endogenous and exogenous lipids are therefore uploaded and presented to a variety of T-cells (CD4+, CD8+, DN).

**Figure 3:**
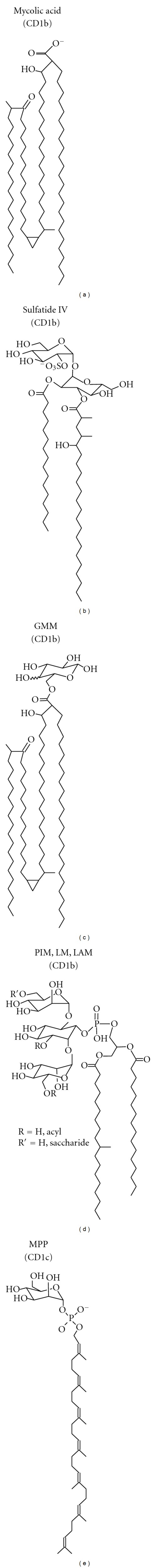
Nonpeptide T-cell antigens associated to the cell wall of *Mycobacterium tuberculosis. *The amphipathic nature of lipids is mandatory for antigen presentation. The variety of lipids associated to mycobacterial cell envelope include mycolic acids, diacylated sulfatide, glucose monomycolate (GMM), phosphatidyl inositol mannosides (PIM), lipoarabinomannan (LAM), and cell-wall biosynthetic precursors from the isoprenoid family, such as mannosyl phosphopolyprenol (MPP). Restriction to specific CD1 isoforms is indicated.

The search for CD1-restricted responses promptly allowed the description of T-cells specific for *M. tuberculosis*. Mycolic acids from mycobacterial cell walls were the first lipid antigens to be described [51]. To date, a broad range of microbial, plant and mammalian self-lipids have been recognized as CD1 antigens. Dideoxymycobactin [52], glucose monomycolate [6, 49], sulfatide IV (SL-IV) [5], isoprenoid lipids [53], phosphatidylinositol mannosides (PIM), and LAM have all been identified as CD1 antigens in mycobacteria. The structural features and restriction of CD1 lipid antigens from mycobacterial cell walls are schematized in Figure 3. Lipid antigens from other microorganisms, including *Leishmania donovani*, *Borrelia burgdorferi,* and *Sphingomonas* have been described as well. Nonmicrobial lipids presented by CD1 include pollen phospholipids that trigger allergy associated mechanisms as well as self-glycolipids involved in autoimmune diseases, including multiple sclerosis [49, 50].

CD1 molecules are very minor components of monocyte-derived macrophages and the study of antigenic lipid presentation implies the use of dendritic cells, which possessed important drawbacks until the work by Sallusto and Lanzavecchia, who described the obtainment of these antigen-presenting cells from blood monocytes [54]. The restricted distribution of CD1 molecules represents a challenge for the study of lipid antigens too (murine animal models of tuberculosis only present the CD1d isoform). Curiously, some CD1d restricted cells share elements of innate immune natural killer cells (NK) and from T-cell receptors (TCR) featured by an invariant *α* chain. These so-called invariant Natural Killer T (iNKT) cells respond very rapidly following *M. tuberculosis* infections. Cognate antigens from mycobacterial origin have not been described yet, but some data support the existence of direct activation mechanisms, namely through cytokines [55, 56]. At present, the involvement of lipids in adaptive immune response is fully recognized, but more studies are needed in order to promote the application of lipid antigenicity for the development of novel tools to combat the disease.

## 6. Conclusive Remarks

The cell-wall of *M. tuberculosis* is rich in carbohydrate and lipid molecules. Recent studies have brought to light the mode of action of important immune modulators from mycobacteria. Both innate and adaptive responses to nonprotein mycobacterial compounds may be exploited for the development of tools to control the disease. Recent reports have advanced the potential of cell wall lipid antigens for vaccine subunits. Namely, Komori and coworkers highlight the activation of T-cells by glucose monomycolate (GMM) [6], whereas sulfatide has been proposed as a candidate for tuberculosis vaccine by Puzo's and de Libero's groups [5, 57]. Given the wide variety of bioactive lipids in the tubercle bacillus, the complete assessment of vaccine candidates should include the study of lipids as potential subunits. In this regard, Asensio and coworkers have shown a correlationship between virulence attenuation in the PhoP-PhoR *M. tuberculosis* mutant, a vaccine candidate, and important alterations in lipid profiles [58]. Interestingly, the cytotoxic response triggered in humans by vaccination with live bacillus Calmette-Guérin has been found to be essentially mediated by CD1 antigens [59]. Nevertheless, there has been a misconception regarding the involvement of CD1 antigen presentation during tuberculosis. Most *in vitro* studies of immune activation have been conducted in macrophages, whereas only dendritic cells are able to present lipids. Similarly, studies addressing the immune response against tuberculosis are commonly performed in mice, a model devoid of four from five human CD1 isoforms. In conclusion, it is to note that the development of new tools for tuberculosis control may take profit of mycobacterial lipids, but more efforts must be undertaken to better elucidate their full potential at both basic and translational fields.

## References

[1] Ryan F (1992). *The Forgotten Plague: How the Battle against Tuberculosis Was Won – and Lost*.

[2] Guenin-Macé L, Siméone R, Demangel C (2009). Lipids of pathogenic mycobacteria: contributions to virulence and host immune suppression. *Transboundary and Emerging Diseases*.

[3] Ehlers MRW, Daffé M (1998). Interactions between *Mycobacterium tuberculosis* and host cells: are mycobacterial sugars the key?. *Trends in Microbiology*.

[4] Dorhoi A, Reece ST, Kaufmann SHE (2011). For better or for worse: the immune response against *Mycobacterium tuberculosis* balances pathology and protection. *Immunological Reviews*.

[5] Guiard J, Collmann A, Garcia-Alles LF (2009). Fatty acyl structures of *Mycobacterium tuberculosis* sulfoglycolipid govern T cell response1. *Journal of Immunology*.

[6] Komori T, Nakamura T, Matsunaga I (2011). A microbial glycolipid functions as a new class of target antigen for delayed-type hypersensitivity. *Journal of Biological Chemistry*.

[7] Goren MB (1972). Mycobacterial lipids: selected topics. *Bacteriological reviews*.

[8] Kresge N, Simoni RD, Hill RL (2008). Chemical investigation of tubercle bacillus lipids: the work of Rudolph J. Anderson. *The Journal of Biological Chemistry*.

[9] Asselineau J, Lanéelle G (1998). Mycobacterial lipids: a historical perspective. *Frontiers in Bioscience*.

[10] Stacey M, Kent PW, Wolfrom ML (1948). The polysaccharides of *Mycobacterium tuberculosis*. *Advances in Carbohydrate Chemistry*.

[11] McNeil M, Wallner SJ, Hunter SW, Brennan PJ (1987). Demonstration that the galactosyl and arabinosyl residues in the cell-wall arabinogalactan of Mycobacterium leprae and Myobacterium tuberculosis are furanoid. *Carbohydrate Research*.

[12] Brennan PJ, Ratledge C, Wilkinson SG (1988). Mycobacterium and other actinomycetes. *Microbial Lipids*.

[13] Hershkovitz I, Donoghue HD, Minnikin DE (2008). Detection and molecular characterization of 9000-year-old *Mycobacterium tuberculosis* from a neolithic settlement in the Eastern mediterranean. *PLoS One*.

[14] Liu J, Nikaido H (1999). A mutant of Mycobacterium smegmatis defective in the biosynthesis of mycolic acids accumulates meromycolates. *Proceedings of the National Academy of Sciences of the United States of America*.

[15] Hoffmann C, Leis A, Niederweis M, Plitzko JM, Engelhardt H (2008). Disclosure of the mycobacterial outer membrane: cryo-electron tomography and vitreous sections reveal the lipid bilayer structure. *Proceedings of the National Academy of Sciences of the United States of America*.

[16] Zuber B, Chami M, Houssin C, Dubochet J, Griffiths G, Daffé M (2008). Direct visualization of the outer membrane of mycobacteria and corynebacteria in their native state. *Journal of Bacteriology*.

[17] Bhamidi S, Scherman MS, Jones V (2011). Detailed structural and quantitative analysis reveals the spatial organization of the cell walls of in vivo grown *Mycobacterium leprae* and in vitro grown *Mycobacterium tuberculosis*. *Journal of Biological Chemistry*.

[18] Abdallah AM, Gey van Pittius NC, DiGiuseppe Champion PA (2007). Type VII secretion—mycobacteria show the way. *Nature Reviews Microbiology*.

[19] Ortalo-Magné A, Lemassu A, Lanéelle MA (1996). Identification of the surface-exposed lipids on the cell envelopes of *Mycobacterium tuberculosis* and other mycobacterial species. *Journal of Bacteriology*.

[20] Rocha-Ramírez LM, Estrada-García I, López-Marín LM (2008). *Mycobacterium tuberculosis* lipids regulate cytokines, TLR-2/4 and MHC class II expression in human macrophages. *Tuberculosis*.

[21] Cywes L, Hoppe HC, Daffé M, Ehlers MRW (1997). Nonopsonic binding of *Mycobacterium tuberculosis* to complement receptor type 3 is mediated by capsular polysaccharides and is strain dependent. *Infection and Immunity*.

[22] Beatty WL, Rhoades ER, Ullrich HJ, Chatterjee D, Heuser JE, Russell DG (2000). Trafficking and release of mycobacterial lipids from infected macrophages. *Traffic*.

[23] Beatty WL, Ullrich HJ, Russell DG (2001). Mycobacterial surface moieties are released from infected macrophages by a constitutive exocytic event. *European Journal of Cell Biology*.

[24] Fujita Y, Okamoto Y, Uenishi Y, Sunagawa M, Uchiyama T, Yano I (2007). Molecular and supra-molecular structure related differences in toxicity and granulomatogenic activity of mycobacterial cord factor in mice. *Microbial Pathogenesis*.

[25] Hunter RL, Armitige L, Jagannath C, Actor JK (2009). TB Research at UT-Houston—a review of cord factor: new approaches to drugs, vaccines and the pathogenesis of tuberculosis. *Tuberculosis*.

[26] Saavedra R, Segura E, Tenorio EP, López-Marín LM (2006). Mycobacterial trehalose-containing glycolipid with immunomodulatory activity on human CD4^+^ and CD8^+^ T-cells. *Microbes and Infection*.

[27] Palma-Nicolás JP, Hernández-Pando R, Segura E (2010). Mycobacterial di-O-acyl trehalose inhibits Th-1 cytokine gene expression in murine cells by down-modulation of MAPK signaling. *Immunobiology*.

[28] Gehring AJ, Dobos KM, Belisle JT, Harding CV, Boom WH (2004). *Mycobacterium tuberculosis* LprG (Rv1411c): a novel TLR-2 ligand that inhibits human macrophage class II MHC antigen processing. *Journal of Immunology*.

[29] Harding CV, Boom WH (2010). Regulation of antigen presentation by *Mycobacterium tuberculosis*: a role for Toll-like receptors. *Nature Reviews Microbiology*.

[30] Vergne I, Fratti RA, Hill PJ, Chua J, Belisle J, Deretic V (2004). *Mycobacterium tuberculosis* phagosome maturation arrest: mycobacterial phosphatidylinositol analog phosphatidylinositol mannoside stimulates early endosomal fusion. *Molecular Biology of the Cell*.

[31] Cole ST, Brosch R, Parkhill J (1998). Deciphering the biology of *Mycobacterium tuberculosis* from the complete genome sequence. *Nature*.

[32] Fournie JJ, Adams E, Mullins RJ, Basten A (1989). Inhibition of human lymphoproliferative responses by mycobacterial phenolic glycolipids. *Infection and Immunity*.

[33] Laneelle G, Daffe M (1991). Mycobacterial cell wall and pathogenicity: a lipidologist’s view. *Research in Microbiology*.

[34] Lopez-Marin LM, Quesada D, Lakhdar-Ghazal F, Tocanne JF, Lanéelle G (1994). Interactions of mycobacterial glycopeptidolipids with membranes: influence of carbohydrate on induced alterations. *Biochemistry*.

[35] Dietrich C, Bagatolli LA, Volovyk ZN (2001). Lipid rafts reconstituted in model membranes. *Biophysical Journal*.

[36] Maldonado-Garcia G, Chico-Ortiz M, Lopez-Marin LM, Sánchez-Garcia FJ (2004). High-polarity Mycobacterium avium-derived lipids interact with murine macrophage lipid rafts. *Scandinavian Journal of Immunology*.

[37] Torrelles JB, Azad AK, Schlesinger LS (2006). Fine discrimination in the recognition of individual species of phosphatidyl-myo-inositol mannosides from *Mycobacterium tuberculosis* by C-type lectin pattern recognition receptors. *Journal of Immunology*.

[38] Torrelles JB, DesJardin LE, MacNeil J (2009). Inactivation of *Mycobacterium tuberculosis* mannosyltransferase pimB reduces the cell wall lipoarabinomannan and lipomannan content and increases the rate of bacterial-induced human macrophage cell death. *Glycobiology*.

[39] Torrelles JB, Schlesinger LS (2010). Diversity in *Mycobacterium tuberculosis* mannosylated cell wall determinants impacts adaptation to the host. *Tuberculosis*.

[40] Torrelles JB, Azad AK, Henning LN, Carlson TK, Schlesinger LS (2008). Role of C-type lectins in mycobacterial infections. *Current Drug Targets*.

[41] Medzhitov R, Janeway CA (1998). An ancient system of host defense. *Current Opinion in Immunology*.

[42] Leulier F, Lemaitre B (2008). Toll-like receptors—taking an evolutionary approach. *Nature Reviews Genetics*.

[43] Jo EK, Yang CS, Choi CH, Harding CV (2007). Intracellular signalling cascades regulating innate immune responses to Mycobacteria: branching out from Toll-like receptors. *Cellular Microbiology*.

[44] Deretic V (2012). Autophagy as an innate immunity paradigm: expanding the scope and repertoire of pattern recognition receptors. *Current Opinion in Immunology*.

[45] Ferwerda G, Girardin SE, Kullberg BJ (2005). NOD2 and toll-like receptors are nonredundant recognition systems of *Mycobacterium tuberculosis*. *PLoS Pathogens*.

[46] Ishikawa E, Ishikawa T, Morita YS (2009). Direct recognition of the mycobacterial glycolipid, trehalose dimycolate, by C-type lectin Mincle. *Journal of Experimental Medicine*.

[47] Kaufmann SH, Hussey G, Lambert PH (2010). New vaccines for tuberculosis. *The Lancet*.

[48] Porcelli S, Brenner MB, Greenstein JL, Balk SP, Terhorst C, Bleicher PA (1989). Recognition of cluster of differentiation 1 antigens by human CD4^−^ CD8^−^ cytolytic T lymphocytes. *Nature*.

[49] Young DC, Moody DB (2006). T-cell recognition of glycolipids presented by CD1 proteins. *Glycobiology*.

[50] Kasmar A, Van Rhijn I, Moody DB (2009). The evolved functions of CD1 during infection. *Current Opinion in Immunology*.

[51] Beckman EM, Porcelli SA, Morita CT, Behar SM, Furlong ST, Brenner MB (1994). Recognition of lipid antigen by CD1-restricted *αβ*
^+^ T cells. *Nature*.

[52] Moody DB, Young DC, Cheng TY (2004). T cell activation by lipopeptide antigens. *Science*.

[53] Moody DB, Ulrichs T, Mühlecker W (2000). CD1c-mediated T-cell recognition of isoprenoid glycolipids in *Mycobacterium tuberculosis* infection. *Nature*.

[54] Sallusto F, Lanzavecchia A (1994). Efficient presentation of soluble antigen by cultured human dendritic cells is maintained by granulocyte/macrophage colony-stimulating factor plus interleukin 4 and downregulated by tumor necrosis factor *α*. *Journal of Experimental Medicine*.

[55] Sada-Ovalle I, Chiba A, Gonzales A, Brenner MB, Behar SM (2008). Innate invariant NKT cells recognize *Mycobacterium tuberculosis*-infected macrophages, produce interferon-*γ*, and kill intracellular bacteria. *PLoS Pathogens*.

[56] Zajonc DM, Kronenberg M (2009). Carbohydrate specificity of the recognition of diverse glycolipids by natural killer T cells. *Immunological Reviews*.

[57] Guiard J, Collmann A, Gilleron M (2008). Synthesis of diacylated trehalose sulfates: candidates for a tuberculosis vaccine. *Angewandte Chemie - International Edition*.

[58] Asensio JG, Maia C, Ferrer NL (2006). The virulence-associated two-component PhoP-PhoR system controls the biosynthesis of polyketide-derived lipids in *Mycobacterium tuberculosis*. *Journal of Biological Chemistry*.

[59] Kawashima T, Norose Y, Watanabe Y (2003). Cutting edge: major CD8 T cell response to live bacillus Calmette-Guérin is mediated by CD1 molecules. *Journal of Immunology*.

